# Supplementary material for: Anterior insular cortex plays a critical role in interoceptive attention
Source: eLife. 2019 Apr 15;8:e42265. doi: 10.7554/eLife.42265 (PMC6488299; doi:10.7554/eLife.42265)
Supplement: Transparent reporting form [file elife-42265-transrepform.docx]

***eLife’s* transparent reporting form**

We encourage authors to provide detailed information *within their submission* to facilitate the interpretation and replication of experiments. Authors can upload supporting documentation to indicate the use of appropriate reporting guidelines for health-related research (see [EQUATOR Network](http://www.equator-network.org/%20)), life science research (see the [BioSharing Information Resource](https://biosharing.org/)), or the [ARRIVE guidelines](http://www.plosbiology.org/article/info:doi/10.1371/journal.pbio.1000412) for reporting work involving animal research. Where applicable, authors should refer to any relevant reporting standards documents in this form.

If you have any questions, please consult our Journal Policies and/or contact us: [editorial@elifesciences.org](mailto:editorial@elifesciences.org).

**Sample-size estimation**

- You should state whether an appropriate sample size was computed when the study was being designed
- You should state the statistical method of sample size computation and any required assumptions
- If no explicit power analysis was used, you should describe how you decided what sample (replicate) size (number) to use

Please outline where this information can be found within the submission (e.g., sections or figure legends), or explain why this information doesn’t apply to your submission:

For fMRI experiment, we chose to include a large sample. For lesion patient study, given the limited and uncontrollable occurrence of patients with focal and particular brain region lesion (the current data was collected over two years), the sample size could not be freely set to a predetermined size.

**Replicates**

- You should report how often each experiment was performed
- You should include a definition of biological versus technical replication
- The data obtained should be provided and sufficient information should be provided to indicate the number of independent biological and/or technical replicates
- If you encountered any outliers, you should describe how these were handled
- Criteria for exclusion/inclusion of data should be clearly stated
- High-throughput sequence data should be uploaded before submission, with a private link for reviewers provided (these are available from both GEO and ArrayExpress)

Please outline where this information can be found within the submission (e.g., sections or figure legends), or explain why this information doesn’t apply to your submission:

The experimental task was performed once for participants in fMRI study (both the 1^st^ sample and the 2^nd^ sample) and for each group in lesion patient study. Data from one participant was excluded from DCM analysis in the first sample because could not identify ROI (see first paragraph in Dynamic causal modeling (DCM) part).

**Statistical reporting**

- Statistical analysis methods should be described and justified
- Raw data should be presented in figures whenever informative to do so (typically when N per group is less than 10)
- For each experiment, you should identify the statistical tests used, exact values of N, definitions of center, methods of multiple test correction, and dispersion and precision measures (e.g., mean, median, SD, SEM, confidence intervals; and, for the major substantive results, a measure of effect size (e.g., Pearson's r, Cohen's d)
- Report exact p-values wherever possible alongside the summary statistics and 95% confidence intervals. These should be reported for all key questions and not only when the p-value is less than 0.05.

Please outline where this information can be found within the submission (e.g., sections or figure legends), or explain why this information doesn’t apply to your submission:

The rationale for different statistical tests is described in the methods of each experiment (e.g. see fMRI data analysis for the first and the second samples; see behavioral data analysis for lesion study).

Results from standard t or ANOVA test are reported with degree of freedom, t or F statistic, and p value, which are embedded in the main text. Patients’ results are presented individually as in Figure 7.

(For large datasets, or papers with a very large number of statistical tests, you may upload a single table file with tests, Ns, etc., with reference to sections in the manuscript.)

**Group allocation**

- Indicate how samples were allocated into experimental groups (in the case of clinical studies, please specify allocation to treatment method); if randomization was used, please also state if restricted randomization was applied
- Indicate if masking was used during group allocation, data collection and/or data analysis

Please outline where this information can be found within the submission (e.g., sections or figure legends), or explain why this information doesn’t apply to your submission:

For fMRI experiment, the human participants were not allocated to experimental groups (i.e., this is a within-subject design). For lesion study, see the section of brain lesion and control groups in the manuscript.

**Additional data files (“source data”)**

- We encourage you to upload relevant additional data files, such as numerical data that are represented as a graph in a figure, or as a summary table
- Where provided, these should be in the most useful format, and they can be uploaded as “Source data” files linked to a main figure or table
- Include model definition files including the full list of parameters used
- Include code used for data analysis (e.g., R, MatLab)
- Avoid stating that data files are “available upon request”

Please indicate the figures or tables for which source data files have been provided:

Source data have been uploaded to Dyrad (<https://datadryad.org/)>, including behavioral data, fMRI data, and lesion patient data. Our DRYAD DOI is: doi:10.5061/dryad.5sj852c. Specifically, for the fMRI study, we uploaded each individual’s contrast maps, fMRI signals of each region of interest, and parameters for model selection and model average in DCM. And for the lesion study, we uploaded behavioral data (i.e. accuracy, d-prime, and beta) for each participant in the groups of normal controls, brain damage controls, and AIC lesions. These data can be used to reconstruct our figures presented in the main manuscript and in the supplemental.
